# Disjunctions between contractual and civil service recruitment: public sector doctors’ perspectives from two Indian states

**DOI:** 10.1186/s12960-025-00990-9

**Published:** 2025-07-18

**Authors:** Bhaskar Purohit, Peter S. Hill

**Affiliations:** 1 Peter Faber Business School, Australian Catholic University, Building 532 – Tenison Woods House, 8-20 Napier Street, North Sydney, 2060 Australia; 2 Faculty of Health, Medicine and Behavioural Sciences, School of Public Health, The University of Queensland, Brisbane, QLD 4006 Australia

**Keywords:** Recruitment, Contractual recruitment, Civil service recruitment, Public service commission, Doctors, India

## Abstract

**Background:**

Civil service and contractual recruitment are common recruitment pathways with significant differences in terms of security and benefits for rural doctors and their career trajectories. However, there are tensions between doctors’ expectations of the systems and the systems’ imaginings of what it offers in terms of recruitment. In this paper, we explore these tensions from the perspective of frontline public sector doctors.

**Methods:**

This qualitative multiple-case study research was carried out in two Indian states. We used semi-structured interviews with 33 doctors and 28 key informants from the two states. Thematic analysis, using the framework approach, was used to arrange and synthesize qualitative data. In addition, job histories were constructed from the doctors’ interviews to examine their experiences with recruitment and analyzed using simple numbers.

**Results:**

The findings suggest that in one study State, the doctors and the administration perceive civil service and contractual recruitment differently with tensions between personal and systemic perspectives. Findings from this State suggest that contractual doctors conceive the progression from contractual to civil service recruitment as sequential. Contrary to doctors, health administration regards these two forms of recruitment as distinct—potentially complementary, but certainly strategic, but not necessarily sequential. However, there are several obstacles faced by doctors that negatively affect their expectations of progressing to civil service recruitment and their career trajectories. The critical obstacles are: prolonged contractual employment, irregular PSC evaluations, and decreasing opportunities to become civil servants. All these factors lead to discontent among contractual employees, with critical consequences for their career trajectories and job satisfaction. However, findings from the other State indicate the use of alternative approaches in recruitment, leading to frequent civil service recruitment and positive perceptions among doctors.

**Conclusions:**

The idea that civil service recruitment forms a continuum with contractual recruitment is a misconception held by rural doctors, while the administration sees important distinctions between them. This disjunction in perspectives is problematic, leading to negative perceptions and breach of doctors’ assumptions, with broader implications. From a health systems and workforce perspective, the need for the administration to acknowledge and address disjunctions through effective human resource approaches is critical.

## Background

The shortage and maldistribution of health workers is a problem shared across many countries, presenting a serious impediment to the achievement of health goals and access to health services [1]. India is no exception to this problem—the country faces critical shortages of doctors, and most of them are skewed in the private sector and urban locations, leading to chronic shortages of doctors in the public sector [2, 3]. The World Health Organization (WHO) acknowledges the complex interplay of several factors contributing to shortages and maldistribution [1, 4]. Most of the Human Resource for Health (HRH) literature and Indian approaches to addressing the shortages center around understanding the issue from a labor market perspective, improving the incentives, and education and regulatory measures [5]. However, little literature is available aimed at understanding the health workforce issues beyond this normal discourse.

The shortages and maldistribution have been described as “symptoms of a poorly managed health workforce and health care system” [6]. This results in the failure of health systems to attract, retain, and deploy workers to the locations, where they are most needed. Thus, addressing the shortages requires effective Human Resource Management (HRM) systems, which is defined by the Global Health Workforce Alliance (GHWA) as the “integrated use of data, policy, and practice to plan for necessary staff, recruit, hire, deploy, develop, and support health workers [7]. Although a strong need exists to understand shortages and maldistribution from an HRM perspective, it has been grossly overlooked [8, 9]. In this paper, we focus on the recruitment of public-sector doctors—one of the most critical aspects of HRM.

The main incentive for doctors to work in the public sector is civil service recruitment—a form of recruitment that offers permanent or ongoing employment with high job security, clear career progression, and employment benefits such as pensions, eligibility for promotion, and salary grades that progress with competence [10]. Civil service recruitment of doctors in India is carried out by the state-specific Public Service Commission (PSC). However, the failure to deploy sufficient civil service doctors throughout the country has led to shortages of doctors in the public sector [11]. To address the shortages, the Government of India initiated a nationwide health systems reform program called the National Health Mission (NHM) in 2005 to complement state civil service recruitment, underwriting 60% of costs with most of the states [12]. The NHM has provided extensive contractual recruitment of allopathic doctors, as well as those trained in alternative systems of medicine, such as Ayurveda, Yoga and Naturopathy, Unani, Siddha, Sowa, Rigpa, and Homoeopathy (AYUSH) [13]. Several Indian states provide opportunities for progression from contractual to civil service recruitment. Despite these efforts, significant shortages of doctors remain—a shortfall of 0.16 million doctors to meet the minimum threshold of 34.5 skilled health workers per 10,000 population [14]. Beyond the numbers, however, there are systemic challenges that make these contractual recruitment initiatives less attractive. These include the vast pay parity, poor career trajectories, growth opportunities, and disparities related to service-related benefits for contractual doctors compared to their civil service counterparts [15]. The consequence is a complex set of competing recruitment pathways, with significant differences in terms of security and benefits for rural doctors and their career trajectories. The HRH literature from India and other LMICs suggests that recruitment of health workers can sometimes be problematic and that both civil service and contractual recruitment can be experienced and perceived negatively by health workers [11, 16, 17]. The negative experiences of recruitment contribute to a lack of motivation, which, in turn, negatively affects attraction and retention, further compounding the shortage of health workers [16].

What is, however, missing in our understanding of recruitment is the complex nature of interplay in the perceptions of different policy actors about recruitment pathways. Understanding the disjunctions in perceptions is vital, because these are potential roadblocks to achieving effective human resource approach that balances individual and organizational goals [18]. More specifically, to date, literature has done little to examine the recruitment-related experiences of public sector doctors, focusing on understanding the disjunctions in the perceptions of frontline public sector doctors and health administration about civil service and contractual recruitment. In this paper, we examine public sector doctors’ experiences with recruitment from two Indian states with a particular focus on understanding the tensions between doctors’ expectations of the systems and the systems’ imaginings of what it offers in terms of recruitment. The context of this exploration is a larger qualitative study to examine public sector doctors’ experiences with the HRM systems from two Indian states. To the best of our knowledge, this is the first HRH paper that unpacks the complexity of doctors'interactions with recruitment, reporting the disjunction in perspectives and thus contributing to the existing literature in two ways. One, it brings to light that the disjunction in perspectives is problematic with broader health workforce and system implications. Second, the paper expands the existing discussion of the need to understand health workers'interactions with state HRM systems beyond the normal discourse of how HRH issues have been researched. This is relevant for understanding and implementing context-appropriate recruitment policies and practices towards a more inclusive and systematic approach to health workforce issues.

**Fig. 1:**
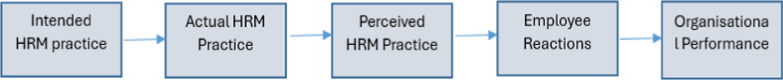
HRM causal chain. Adapted from Boxall, P. and K. Macky (2007)

The theoretical framework that underpins our research is the HRM causal chain theory, which focuses on HRM performance and its underlying causal chain [19]. This framework is explained by representing the HRM process as a linked chain—where the intended recruitment policies and practices are translated into actual practices (see Fig. 1). Any gaps between actual policies and practices and how employees perceive these may lead to employee reactions and, collectively, in organizational performance [19]. In this paper, we specifically focus on the second and third boxes (actual recruitment practices and how doctors and health administration perceive these), while other aspects of the framework, such as employee reactions, are explored in a separate paper.

### Recruitment context

In the Indian context, recruitment is used as a broad term with three main recruitment forms that describe the nature of employment: civil service recruitment (with indefinite tenure), ad hoc recruitment, and contractual recruitment (with fixed tenure). Recruitment rules guide civil service recruitment in each State, detailing requirements for specific posts, various methods of recruitment (direct recruitment, promotion, and deputation), and the eligibility criteria for each post. Recruitment and selection by PSCs may depend upon the number of government-approved sanctioned posts, the availability of doctors, the number of applications, and state-specific recruitment rules [20]. Each state’s PSC conducts civil service examinations and/or interviews and prepares a list of selected candidates based on ‘merit-cum-seniority’, which states use to determine the actual date of civil service recruitment and employment-related benefits.

The two other types of recruitment are ad hoc and contractual recruitment. Ad hoc recruitments preceded the NHM program and were used to fill vacant positions temporarily, with unspecified tenure, and with the policy imperative that all sanctioned posts need to be filled under civil service recruitment. Following the introduction of the NHM in 2005, a recruitment and selection process involving *face-to-face interviews* and/or theory exams has offered fixed-term, 1-year contractual appointments, depending on available positions and the number of applicants.

The processes and agencies for civil service and contractual recruitment of doctors in the two study states are mainly similar. The PSCs from the two study states are the central bodies responsible for the civil service recruitment of doctors, and similarly, the contractual recruitment of allopathic and AYUSH doctors is standard across both study states. In this paper, we use ‘civil service recruitment’ as a descriptor for those doctors recruited under permanent employment. Respondent quotes, however, may refer to civil service recruitment as'regular recruitment or regularization.'

## Methods

### Study design and setting

This research was conducted in two Indian states using a qualitative case study approach. The two states were purposively selected as distinct cases for the socioeconomic diversity, differences in health workforce indicators such as number of regular and temporary doctors in public service, the considerable variances in the public health infrastructure such as number of medical colleges, District Hospitals (DH), Community Health Centres (CHC), and Primary Health Centres (PHC), and the differences in other health indicators such as Under-5 and IMR. For research purposes, the state names are anonymized and referred to as State Y and Z. State Y is one of the smallest states in India by geographic area. The Infant Mortality Rate (IMR) of the State was 30 per 1000 live births, and the Under-5 (U-5) mortality rate was 32 per 1000 live births [21]. The State has one state referral hospital, four DHs, two CHCs, and 24 PHCs. In contrast, State Z is among the largest states by geographic area. Nearly, three quarters (75.13%) of the State's population lives in rural areas. The IMR of the State is 41 per 1000 live births, and the U-5 mortality rate is 51 per 1000 live births [21]. The State has 33 districts with eight government medical colleges, 34 DHs, 12 SDHs, 588 CHCs, and 2078 PHCs [22].

### Study participants and data collection

To understand doctors'recruitment-related experiences, we used semi-structured interviews with 33 frontline doctors—15 from Y and 18 from Z. Purposive sampling with maximum variation was used to include doctors from the two states. As Y is a small state, we had representation of doctors from all four districts in this State. However, for Z, we used purposive sampling to interview 18 doctors representing six districts who were purposively selected to ensure the representation of doctors from districts that were close to and remote from state headquarters.

In addition, to understand the existing recruitment systems, we interviewed 28 Key Informants (KIs)—16 from Y and 12 from Z. Purposive sampling was used in both states to identify KIs based on their knowledge about the research topic, the institutions, and the positions they held in various institutions, ensuring a wide representation of key policy actors.

The lead author (BP) carried out the data collection from February to October 2019. Interviews with participants were conducted at their workplaces, except for six interviews with doctors conducted at a training centre. The interview transcripts for all the interviews were imported into NVivo 12 for analysis [23].

### Data analysis

We used Framework Analysis (FA), a matrix-based method, to arrange and synthesize qualitative data based on the themes generated from the data [24]. FA was particularly suited to the current research for several reasons. One, the current research uses an integrated approach (with a combination of both inductive and deductive analysis), which is a main feature of FA [25]. Two, FA offers flexibility in its adaptation to any qualitative methods that aim to generate themes [25]. Third, FA is a useful method to capture diversity around a phenomenon [26]. The current research identified several themes (A priori and emerging themes) that capture the interactions and experiences of doctors with recruitment. These themes capture diverse personal experiences across the two cases and between regular and contractual doctors, such as doctors’ expectations, perceptions, and experiences of different recruitment pathways, as well as the conflicting perceptions between doctors and administration. Thus, FA was deemed suitable. For the analysis, the data were organized and synthesized by coding the text, grouping the coded texts, and synthesizing the meaning based on the A priori and emerging themes. The themes thus developed were applied to all transcripts using NVivo 12. In addition, we constructed job histories from the doctors’ interviews to examine their experiences with the recruitment system. The quantitative data, captured through job histories, were analyzed using frequencies and triangulated with the narrative accounts given by doctors.

## Findings

### Participants’ profile

The key demographic and work-related characteristics of the 15 doctors from State Y and 18 from State Z are given in Table 1. The study also included the following 28 KIs (16 from State Y and 12 from State Z): three state-level bureaucrats; four state-level health administrators; five district-level health administrators from the Department of Health; three senior administrative officers from the NHM; three administrators from the AYUSH Department; four doctors from the States’ Medical Officer Association; one representative from the public service commission; and five other KIs from other institutions.

**Table 1:** Demographic and work-related profile of the doctors

	State Y			State Z		
	Civil service allopathic n = 5	Contract allopathic n = 6	Contract AYUSH n = 4	Civil service allopathic n = 14		Civil service AYUSH n = 4
	States’ PSC			States’ PSC	Other Agency	
Age						
Mean	44 (40–48)	30.33 (27–34)	28(27–29)	45.8 (31–57)	30.5 (27–37)	44.25 (35–51)
Sex						
Male	4	4	0	5	7	4
Female	1	2	4	2	-	-
Total	5	6	4	7	7	4
Current place of work						
PHC	2	4	2	1	7	2
CHC	1	2	1	5	–	1
SDH/DH	2	–	1	1	–	–
Dispensaries	–	–	–	–	–	1
Total	5	6	4	7	7	4
Total service (in months)						
Mean (range)	216 (156–264)	54 (8–120)	42 (24–60)	221.1 (48–396)	36 (12–60)	169.5(24–276)
Current place (in months)						
Mean (range)	57.6 (36–84)	24 (8–60)	34(4–60)	69.4(30–108)	22.5 (6–36)	46.5 (18–84)

Our analysis of the qualitative data and the job histories is captured through the following themes:

1. Doctors’ expectations that civil service recruitment will be achieved
2. Infrequent PSC civil service recruitment in the two states
3. Consequences of infrequent PSC recruitment
4. Doctors'perceptions and experiences of states’ responses to infrequent PSC recruitment
5. Conflicting perceptions between doctors and administration in State Y

### Contractual doctors’ expectations that civil service recruitment will be achieved

Several contractual doctors indicated that as they progressed through the service, they had strong expectations of enhanced opportunities for civil service recruitment. Some of these doctors also presumed that the period they worked as contractual employees would count during the service period and boost their chances for civil service recruitment.

When I joined on contract, I thought I would have the chance to apply for PSC soon. (State Y, contractual doctor 14)

I have continued in the service for the last five years because I hoped that I would be regularized at some point… Some of my friends [referring to contractual doctors], working in other states have had opportunities to get into regular jobs so I expected the same for myself. (State Y, contractual doctor 12)

However, there were several obstacles faced by doctors that affected their expectations of progressing to civil service recruitment and their career trajectories negatively.

### Infrequent PSC civil service recruitment in the two states

Doctors from the two states reported the failure of the PSC to conduct scheduled evaluations. In State Z, doctors appointed as *ad doc* recruits (before 2012) reported experiencing infrequent PSC, with its infrequent civil service recruitment primarily attributed to the high workload of the State’s PSC.

There was a delay in the exams when PSC was conducting it, as there was too much workload on them. It sometimes took more than 2–3 years. (State Z, Civil Service Doctor 02)

Doctors and KIs from State Y reported much longer gaps (nearly 10 years) in the conduct of PSC civil service recruitment compared to State Z.

The state public service commission has not conducted interviews for regular posting for the last 10 years. (State Y, KI 07)

I think there has been no PSC selection in the last 10 years. (State Y, Contractual doctor 05)

### Consequences of infrequent PSC recruitment

Infrequent PSC recruitment, especially in State Y, contributed to longer periods served on contracts, limited avenues for promotion and other service-related benefits, and the diminishing likelihood of being upgraded to civil services.

### *Long duration served on *ad hoc* service and its implications:*

In states Y and Z, doctors reported long periods of working as ad hoc recruits—from starting employment with the Department of Health to when these doctors cleared civil service recruitment through the PSC. The average time worked ad hoc varied for the two states (see Table 2).

I worked for five years on ad hoc and after five years I was regularized as there was no PSC. (State Y, Civil Service doctor 02)

I joined the service in 1987, but as the State’s PSC had not issued vacancies for doctors, I got into regular service in 1992. (State Z, Civil Service doctor 01)

**Table 2:** Period served as ad hoc before civil service recruitment through PSC in the two states

State	Period served as ad hoc before getting into civil service in months (*A*)	Total service in months with the Department of Health (*B*)	Proportion of time to get into civil service (from the time of joining) relative to total service (*A*/B * 100)
State Y (doctors recruited through PSC)			
Doctor 1	2	156	1.28
Doctor 2	60	240	25
Doctor 3	2	264	0.75
Doctor 4	36	156	23.07
Doctor 5	24	264	9.09
State Y: total (mean)	124 (24.8)	1080 (216)	(11.83)
State Z (doctors recruited through PSC)			
Doctor 1	60	360	16.66
Doctor 2	24	276	8.69
Doctor 3	5	396	1.26
Doctor 4	6	120	5
Doctor 5	7	220	3.18
Doctor 6	1	54	1.85
Doctor 7	1	84	1.19
State Z: total (mean)	104 (14.85)	1510 (215.71)	(5.40)
State Y and Z: total (mean)	228 (19)	2590 (215.83)	(8.61)

Job history data for the twelve ad hoc doctors indicate that they spent large proportions of their total service duration as ad hoc appointees. Three doctors from each state indicated that the service period worked as ad hoc and did not count towards civil service, which negatively affected their avenues for promotion and other service-related entitlements.

For the 5 years, I worked as ad hoc, and this period was not counted toward service regularization or anything. Only the period after I was selected by PSC was counted towards service benefits. (State Z, Civil Service doctor 01)

### Longer periods served as contractual recruits and its implications

The job histories of all ten contractual doctors from State Y illustrate that contractual doctors spent much longer periods (mean of 50 months) as contractual recruits (see Table 3) compared to their ad hoc counterparts (mean of 19 months) (see Table 2). Almost all contractual doctors attributed the prolonged periods of work as contractual recruits to the unusually long gap in the conduct of the PSC.

I did not have any opportunity to apply for regular service as there has been no PSC in ten years. (State Y, Contractual doctor 05)

**Table 3:** Period served on contracts (up to the time of interviews) for doctors in State Y

Number	Recruiting agency	Type of practice	Period (in months) worked on contractual
1	NHM	Allopathic	96
2	NHM	Allopathic	8
3	NHM	Allopathic	120
4	NHM	Allopathic	24
5	NHM	Allopathic	60
6	NHM	Allopathic	24
7	NHM	AYUSH	60
8	NHM	AYUSH	24
9	NHM	AYUSH	48
10	NHM	AYUSH	36
Total (mean)			500 (50)

Two KIs highlighted the merits of contractual recruitment as it helps them with quick recruitment and fill required vacancies.

Contractual appointment helps the administrators as we can quickly recruit and fill up the required vacancies. (State Y, KI 08)

### Uncertainty and diminishing likelihood of being upgraded to a civil servant

Contractual doctors from State Y highlighted the uncertainties associated with PSC evaluation, with significant consequences, such as diminished job security, poor career progression, and reduced avenues for service-related benefits.

We want some security and a secure job. There has been no regular recruitment for so many years, and we don’t know when the next PSC recruitment will be, so we feel insecure. (State Y, contractual doctor 01)

I have served for over nine years, but when I get regularized in the future, I have to start from scratch. All the years I have worked as a contractual doctor will not count toward promotion and pension. (State Y, contractual doctor 03)

Due to the unusually low frequency of PSC, many contractual doctors highlighted the importance of PSC recruitment and expressed their wish to appear in the next PSC selection whenever it might be offered.

I think PSC will be conducted next year after probably 10 years. Of course, I am going to apply for PSC when it is offered as it’s a golden opportunity. Probably they might not conduct that again. (State Y, Contractual doctor 05)

### Demotivation to work

The contractual doctors from State Y brought up issues around their discontent with the existing recruitment arrangements, although most were not explicit about how it affected their motivation. However, two doctors explicitly highlighted their frustrations with the long duration served on contracts and how this demotivated them.

Honestly, I have very little motivation to work now. Tell me, which doctor would have the motivation knowing that neither their service period on contract will ever be counted toward promotion, but more importantly when there are great uncertainties for regularization. (State Y, contractual doctor 03)

### Doctors'perceptions and experiences of states’ responses to infrequent PSC recruitment

The two states’ responses to infrequent PSC recruitment were very different. In State Z, doctors and the KIs reported that due to the overburdened State PSC, another agency (name anonymized and referred to as ABC) was delegated the responsibility of civil service recruitment of doctors from 2012 onwards. Most doctors from State Z reported this as a positive step that led to more frequent conduct of civil service recruitment in the State. The job histories of doctors recruited through the ABC suggest exceedingly reduced periods served ad hoc compared to those recruited through the PSC in the two states (see Table 4).

Recruitment through the ABC body happens almost yearly, and it’s also very quick. (State Z, Civil Service doctor 04)

**Table 4:** Comparison of duration worked as ad hoc for doctors recruited through PSC and ABC

Number	Recruiting agency	Period (in months) worked on ad hoc before PSC recruitment
1	State Y PSC	2
2	State Y PSC	60
3	State Y PSC	2
4	State Y PSC	36
5	State Y PSC	24
Mean		24.8
6	State Z PSC	60
7	State Z PSC	24
8	State Z PSC	6
9	State Z PSC	5
10	State Z PSC	7
11	State Z PSC	1
12	State Z PSC	1
Mean		14.85
13	State Z ABC	1
14	State Z ABC	0.5
15	State Z ABC	3
16	State Z ABC	5
17	State Z ABC	2
18	State Z ABC	5
19	State Z ABC	4
Mean		2.92

By comparison, doctors in State Y perceived that the State's administration has continued to rely on contractual recruitment for several years, further compounding gaps in PSC evaluation in the State.

Yes, the slow recruitment under the PSC has affected regular appointments. Because of this, almost 90% of the doctors working in the PHC or even districts are NHM contractual doctors. (State Y, Civil Service doctor 07)

In State Y, there was discontent among contractual doctors that things had progressively worsened with PSC recruitment. This is evident from the job history data—ad hoc recruits in State Y transitioned to civil services much quicker (mean of 24.8 months, see Table 2) than those recruited on contracts (mean of 50 months, see Table 3). Furthermore, contractual doctors from State Y were yet to secure civil service recruitment up to the time of interviews due to no PSC evaluation in the State for the last 10 years.

### Conflicting perceptions between doctors and administration in state Y

There were discrepancies between doctors'perceptions and the administration's justification of reasons for infrequent PSC recruitment. From the administration's viewpoint, the primary reason for not conducting PSC evaluations was insufficient government-sanctioned posts for civil service doctors. However, from the doctors'viewpoint, the State's over-reliance on recruiting NHM contractual doctors resulted in a situation, where most doctors recruited at rural health centres in the State are contractual doctors.

Doctors perceived the State's strategy to continually recruit NHM doctors and not provide opportunities for civil service recruitment as a tactical approach. Doctors reported that this approach allows the State to limit its expenditure in dual ways. It allows the State to avoid paying high salaries and other service-related benefits available to civil service doctors. The State can also limit its salary expenditure for NHM contractual doctors through the significant subsidies offered by the central government.

We have limited sanctioned posts, so this was the hindrance, and we did not have recruitment for the last 10 years. (State Y, KI 08)

I think why PSC has not been offered regularly is because our health department was so relaxed after the introduction of the NHM. They have not felt the need to recruit any regular doctor because the NHM was fulfilling the needs, and the central government is giving money. (State Y, contractual doctor 10)

While the health administration saw contractual recruitment as a temporary solution to meet the requirements of doctors in the State, doctors perceived this as a routine, permanent feature of the system.

We understand that contractual appointment is not a perfect solution … it only helps the administrators to provide a short-term solution. (State Y, KI 09)

I think there has been no PSC since 2008, so the government has kept recruiting under the NHM. We have NHM which is already in place and funding the doctors, so what is the need for regular recruitment? (State Y, Civil Service doctor 04)

## Discussion

In this study, we explored public sector doctors’ experiences with recruitment from two Indian states. The analysis suggests that the two forms of recruitment (civil service and contractual) are perceived differently by the doctors and the administration, with tensions between the personal and systemic perspectives. The State regards recruitment very differently compared to the doctors—the State has some equanimity about it but no commitment to one over the other. The health administration regards these two forms of recruitment as distinct—potentially complementary, but certainly strategic, but not necessarily sequential. In contrast, the doctors appointed on contracts conceive the progression from contractual to civil service recruitment as sequential; failure to progress is seen as inequitable. These doctors perceive the states’ actions as apathetic, calibrated, and measured strategies of the administration that sabotage their hopes and expectations of progressing to civil service recruitment. The difference between these perceptions leads to situations, where doctors struggle to accept their failure to progress to civil service recruitment, the significant consequences for their career trajectories, and their job satisfaction.

According to the study framework, the paper highlights how the doctors understand and accept the intended recruitment policy and practice—they initially accept the imperatives of ad hoc and contractual recruitment to gain entrance to state services. This acceptance is qualified by strong assumptions and expectations that this will lead to civil service recruitment. These assumptions and expectations are partly reinforced by the existing institutional and policy environment around recruitment in other states. However, the actual recruitment practices are experienced differently from the rhetoric and doctors’ expectations, creating implementation and expectation gaps. Some of the gaps highlighted in the study relate to prolonged contractual employment and irregular PSC evaluations, with this trend worsening in State Y and decreasing opportunities to become civil servants in State Y. Thus, another part of the HRM causal framework that our research reinforces is the links between doctors'experiences with recruitment and their negative perceptions and discontentment.

Although we did not analyze doctors'reactions in the current research, our paper provides valuable insights into understanding how unmet expectations lead to negative perceptions and employee work attitudes like lack of motivation. These findings have important practical implications from an implementation perspective, as the HRH literature supports that doctors'unmet expectations and negative perceptions can drive strong negative reactions. Studies show that doctors accept service requirements as imperative for their first appointment [27]; however, as they gain more experience in the system, their own professional and personal needs gain dominance, which drives their behaviors, and they engage in political actions to influence their postings [28]. In addition, considering the Indian landscape, where recruitment-related matters around the employment conditions of doctors have been legally challenged [29]. These suggest a strong need for the administration to be conscious of potential strong actions from doctors with unmet expectations.

Due to study design limitations, we did not analyze the organizational performance. Nonetheless, our current study highlights a few implicit aspects of health systems performance. This paper highlights how negative perceptions lead to demotivation and discontent among doctors. The consequences of infrequent PSC for ad hoc and contractual doctors highlighted in this study are corroborated by another research from India reporting the consequent denial of employment benefits for ad hoc doctors, causing dissatisfaction and demotivation [29]. More importantly, though, the paper highlights how certain system inefficiencies like slow recruitment lead to a circular problem—these slow down and affect the number of doctors appointed to civil service, thus leading to more contractual recruitment. This only compounds the health workforce issues as the lack of access to civil service recruitment was identified as a critical determinant of doctors’ discontent and negative perceptions. This has critical implications for health workforce retention. Several studies from LMIC settings, such as India and Bangladesh, have reported infrequent PSC recruitment [16, 17, 30, 31] and its implications for health workers’ motivations and intentions to discontinue their jobs [16].

Despite the merits of the HRM causal chain framework, there is one limitation. While the framework may be helpful in examining HRM systems that are simple, logical, and sequential, such as those in small to medium-business settings [32], it does not adequately capture the complex nature of the disjunctions between doctors’ (personal) and health administration (systemic) perceptions. The inability of the State to recognize the tensions between systemic and personal issues is a novel finding from the paper, which has significant consequences that are critical from the health workforce and management perspectives. First, this leads to negative perceptions among doctors who believe infrequent civil service recruitment and frequent contractual recruitment are tactical approaches. Second, it negatively impacts doctors’ career trajectories, potentially affecting employees'trust and job satisfaction.

This study results show that the administration views appointing staff on a contractual basis as a strategic but temporary solution to meet the health system's needs. Due to the requirements of the national health program in India, contractual recruitment practices exist in many Indian states in parallel to civil service recruitment [29]. Contractual recruitment has merits—these have been documented to address the overall availability of health workers at public health facilities [33], and health administrators see it as a tool to ensure accountability, less absenteeism, and better workforce performance, and a more flexible option to prioritize rural and remote postings in India [5]. However, the claims from doctors reveal a contrasting picture as they believe that their appointment on contracts is a cost-cutting strategy, supported by two reasons: One, the contract model has been documented to be used to reduce government expenditure [34]. And two, the NHM is, in part, a centrally funded program, and for State Y, the fund-sharing pattern between the central government and the state governments is 90:10 [35]. Literature supports that the contractual model has been linked to high attrition and can lead to large vacancies at a single point, especially when contractual recruitment is carried out in large numbers to compensate for the lack of civil service recruitment [29]. Our findings also suggest the dominance of State Y in relying continually on contractual recruitment, and this status quo is perceived as unfair by contractual doctors, with critical implications on career trajectories and health workforce implications. Despite such problematic health workforce issues, the State has retained an inefficient relationship with its PSC and doctors. Addressing these disparities is critical, as insecurity and lack of career progression have been reported to be demotivating factors among health workers in Sierra Leone [36] and India [16, 37–39]. Unresolved health workforce issues can lead to distrust, dissension, and conflicts in the health sector [23] and protests among health workers [40]. Although the recruitment of contractual staff may be a logical component of an HR strategy, this study highlights how the disjunctions in perceptions of health administration and doctors can be problematic and why State Y should not use contractual appointments as an alternative recruitment approach to avoid the civil service recruitment and selection regulations [34]. The health administration must acknowledge some of these disjunctions to create positive perceptions that would improve motivation, trust, job satisfaction, and doctors'availability. Thus, we recommend a more proactive approach from the health administration to understand any perceived inequities of access to civil service recruitment and acknowledge some disjunctions to create positive perceptions. From an HRM perspective, this can lead to improved congruence between management’s intentions, articulation, and practice of HR policies and how these are perceived by employees, like in the case of State Z.

State Z recognizes and addresses the tensions between systemic and personal issues by better articulating HR approaches with positive implications. It has been achieved by delegating recruitment to ABC, leading to positive perceptions among doctors and addressing the bureaucratic bottlenecks associated with recruitment, especially when civil service recruitment can be slow, rigid, and complex [41]. Another research study from the same State reported that recruitment through the ABC reduced the turnaround time for recruitment between 3 months and 1 year [42]. These findings suggest an alternative and effective HR approach that goes beyond the envisaged conservative role of the PSC to address recruitment-related bottlenecks. There are a variety of other recruitment practices used across Indian states, such as the recruitment of contractual doctors for up to 3 years instead of 1 year [5] and the appointment of doctors on contracts in the State Z; however, only for specific periods to respond to state emergencies [43]. Such initiatives and practices, however, are not consistent across the country. Similarly, alternative HR approaches have been adopted in some Indian States to address challenges resulting from conflicting tensions between the health workforce and administration. These include more opportunities for civil service recruitment, more frequent recruitment in other Indian states, and greater transparency in deployment policy and its applications in the State of Tamil Nadu [44].

In summary, the current research highlights several important aspects of HRM. First, it emphasizes that differences between the'rhetoric'and the'reality'may lead to implementation and expectation gaps. As reported in the paper, the gaps allow researchers to understand how these lead to negative perceptions, which are critical to examining the effectiveness of the HRM systems. The second vital point is that if the health systems want to achieve desired outcomes, such as addressing doctors'shortages and their inequitable distribution, it is important to acknowledge any discordance between perceptions, which could lead to improved HR outcomes. Finally, we recommend the development of a more robust framework that can capture the complex nature of tensions between health workers and management. The topic needs acknowledgment from researchers that recruitment is a complex process. This topic needs a better understanding of organizational complexities, such as the interplay of management's and employees'perspectives, to achieve effective HRM [45].

This study has a few limitations. There are constraints in the sample, with a limited number of doctors interviewed from the two states, which limits the generalizability of the study findings. While the study findings about the infrequent conduct of PSCs resonate with those of other Indian states, generalizability becomes a challenge due to the diverse nature of states in India, where recruitment remains a state matter. The other limitation is recall bias, which is the inability to specify exact dates for job-related events related to recruitment, such as the timing of PSC examinations.

## Conclusion

The idea that civil service recruitment forms a continuum with contractual recruitment is a misconception held by rural doctors, while the administration sees important distinctions between them. This disjunction in perspectives is problematic, leading to negative perceptions and breach of doctors’ assumptions, with broader implications. From the administrative perspective, recruitment of temporary staff may be a logical component of an HR strategy, but the doctors’ experiences suggest that it should not be used continually as an alternative strategy to avoid civil service recruitment. From a health systems and workforce perspective, the administration must acknowledge disjunctions and adopt effective HR approaches such as regular opportunities for civil service recruitment, the creation of institutions, and effective delegation to address system bottlenecks is critical.

## Data Availability

No datasets were generated or analysed during the current study.

## Abbreviations

AYUSH: Ayurvedic, Yunani, Siddha and Homeopathy
CHC: Community Health Center
DH: District Hospital
HR: Human Resource
HRH: Human Resource for Health
HRM: Human Resource Management
KIs: Key Informants
LMIC: Low–Middle-Income-Countries
PHC: Public Health Centre
PSC: Public Service Commission
NHM: National Health Mission
WHO: The World Health Organization
